# **The Therapeutic Effects of Intracavernosal Plaque Excision in Peyronie’s Disease: A None Grafting or Tunical Excising Procedure**

**Published:** 2016-01

**Authors:** Hassan Ahmadnia, Ali Kamalati, Mehdi Younesi Rostami, Mohammad Mehdi Imani, Amir Abbas Asadpour, Mohammad Kazem Hariri

**Affiliations:** 1 Department of Urology, Faculty of Medicine, Mashhad University of Medical Sciences, Mashhad, Iran;; 2 Department of Urology, Faculty of Medicine, Kerman University of Medical Sciences, Kerman, Iran;; 3 Department of Urology, Faculty of Medicine, Mazandaran University of Medical Sciences, Sari, Iran

**Keywords:** Penis, Peyronie’s disease, Intracavernosal plaque, Excision

## Abstract

**BACKGROUND:**

Current surgical treatments in Peyronie’s disease are accompanied by complications such as penile shortening, loss of sensation, erectile dysfunction and recurrence of disease. The aim of this study was the evaluation of clinical results of intracavernosal plaque excision in Peyronie’s disease.

**METHODS:**

The operation was performed on 35 men. It was consisted of incising the tunica albuginea parallel to the plaque and through this incision, and the plaque was removed from the inside surface without excision or replacing the underlying tunica albuginea by grafts. All patients were evaluated before and periodically within 12 months after the surgery with measurement of penile length, curvature angle in the rigidity phase, and sexual satisfaction.

**RESULTS:**

The mean age of patients was 51.4±5.3 years (range 42-59 years). The angle of penile curvature was 25-45° (mean=35°). Thirty patients (86%) obtained a nearly complete straightening of penis. All patients restored their previous penile length without any disorder of sensation within the glans penis and expressed improvement of sexual activity.

**CONCLUSION:**

Intracavernosal plaque excision is a simple, easy and minimal invasive method that does not result in penile shortening, loss of sensation or erectile dysfunction. In properly selected patients, this technique can lead to acceptable elimination of penile curvature and sexual satisfaction.

## INTRODUCTION

Peyronie’s disease is characterized by the formation of plaque or indurated areas in the tunica albuginea of the corpora cavernosa. The tunica albuginea is bilaminar throughout most of its circumference and is composed of an inner circular layer and an outer longitudinal layer. Many authors note that in the case of Peyronie’s disease, calcified plaques seems to involve the inner circular lamina and can be removed, leaving the outer lamina intact.^1^

The plaque is usually located on the dorsal surface of the penis and sometimes causes significant dorsal curvature. Peyronie’s disease often results in painful erection, penile deformity, erectile dysfunction, and serious complications in sexual wife.^2^ The true prevalence of disease is unknown, although some studies have shown prevalence rates ranging from 1% to 4% which is the highest at the ages of 40–60 years.^3^

The disease has two phases in most patients; an early active phase associated with painful erections and changing deformity of the penis, and a later quiescent phase in which the pain disappears and the deformity is stabilized. Conservative medical management is the initial treatment of choice for patients with active phase disease. A variety of medications has been utilized including vitamin E, potassium aminobenzoate (Potaba), colchicine, tamoxifen, and carnitine. In addition to oral therapy, intralesional injections of verapamil and collagenase and various forms of energy such as extracorporeal shock wave therapy (ESWT) and transdermal electromotive administration (iontophoresis) have been applied for the treatment of Peyronie’s disease.^3^

Few medical methods have been proven effective in large clinical trials**. **Surgical correction is the treatment of choice when the deformity or erectile dysfunction precludes intercourse, but should not be considered until the disease has reached its stable and mature phase.^3^ Several surgical procedures have been used for correction of penile curvature that every of them have advantages and disadvantages. Treatment of Peyroni’s disease by the intracavernosal plaque excision is a new surgical technique. The outcomes of this new interesting operation method have published only on 16 patients.^4^ Here we represent our results of the use of this technique for the surgical correction of Peyronie’s disease in our series. 

## MATERIALS AND METHODS

Intracavernosal plaque excision was performed in 35 males, referred to Ghaem Urology Clinic between 2009 and 2012. The diagnosis of Peyronie’s disease was based on a palpable penile plaque and acquired penile curvature. The basic criteria for selecting the patients for operation were a Peyronie’s plaque that is not response to current medical treatments, duration of the disease no shorter than 12 months, a stable state of the disease of at least 6 months, localized lesions, impaired sexual intercourse, and curving during erection that is not due to penile fracture.

**Fig. 1:**
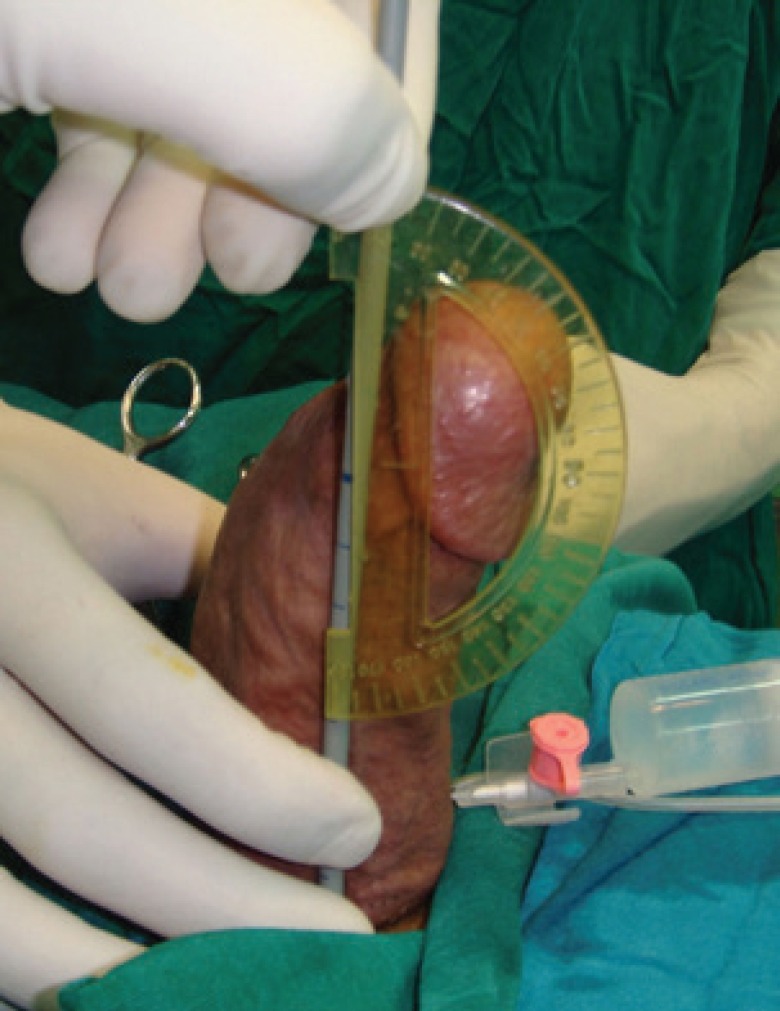
The artificial erection before the surgical correction reveals a dorsal curvature

**Fig. 2:**
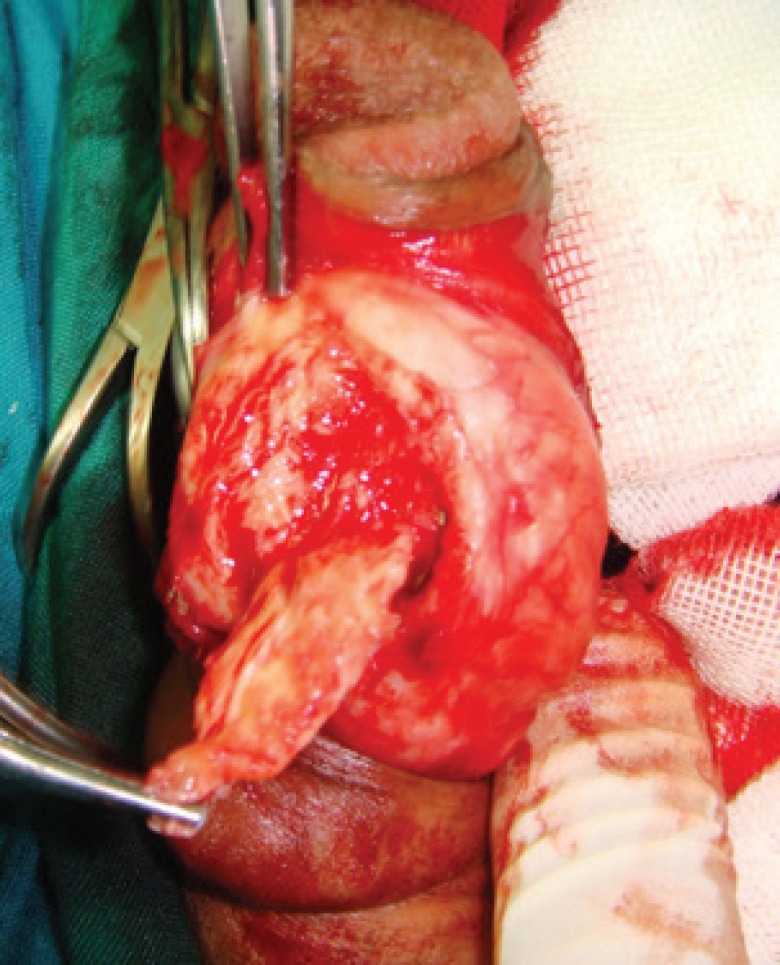
Tunica albuginea is exposed and plaque is identified

The operation was performed in the supine position with general anesthesia. Intraoperative artificial erection was induced with intracorporeal infusion of 0.9% saline to estimate the angle of deviation and penile length (Figure 1). A circumferential subcoronal incision was made and the penis was degloved. Then, buck’s fascia was longitudinally incised and tunica albuginea was exposed and plaque was identified (Figure 2). 

**Fig. 3:**
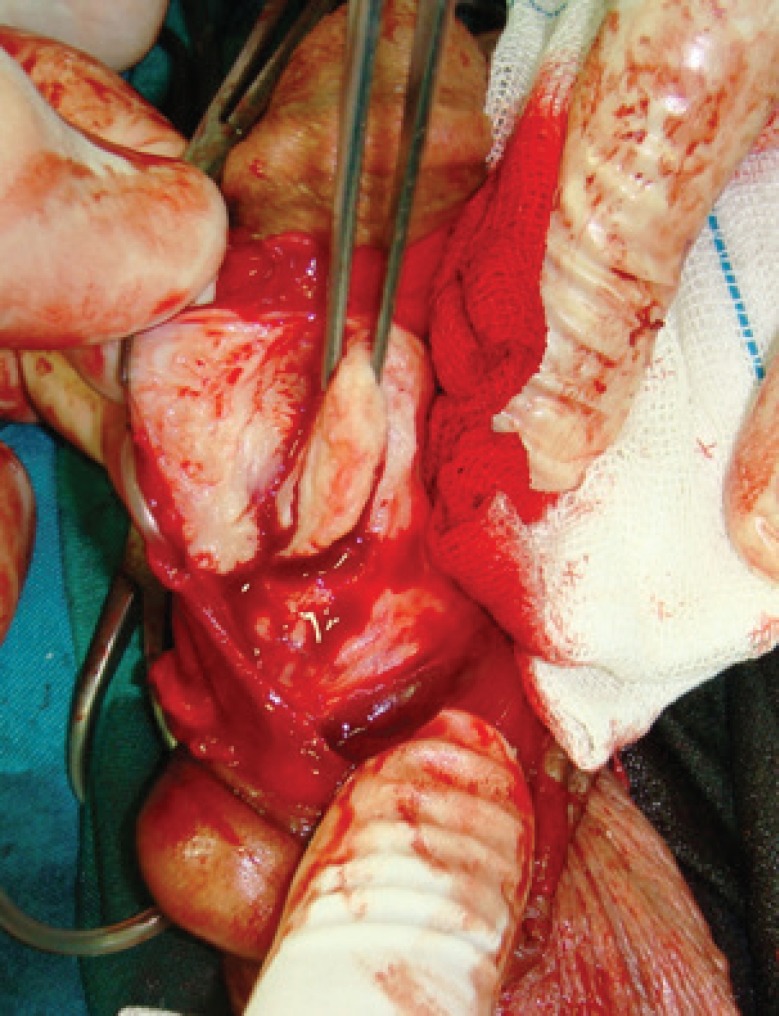
Longitudinal incision of tunica albuginea parallel to the plaque is made

**Fig. 4:**
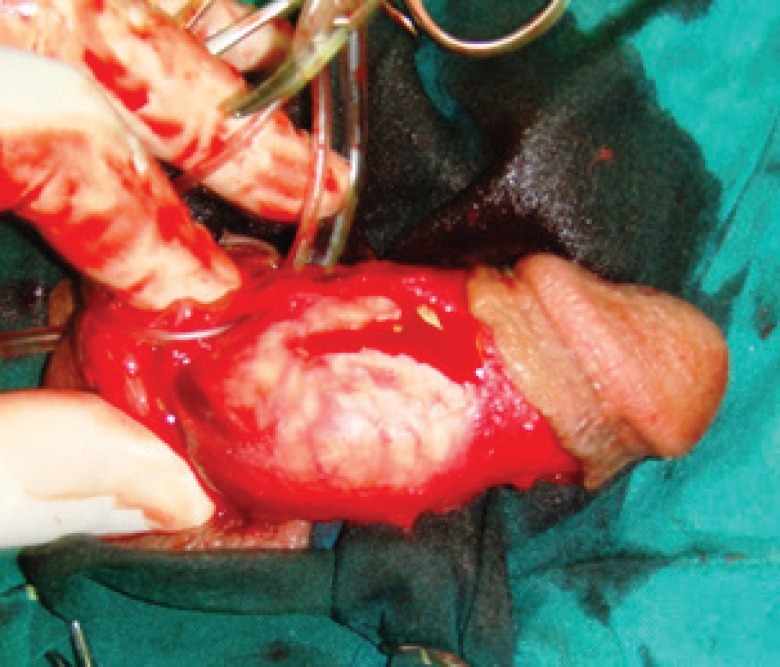
From the inner side of tunica albuginea, the plaque is separated and excised

**Fig. 5:**
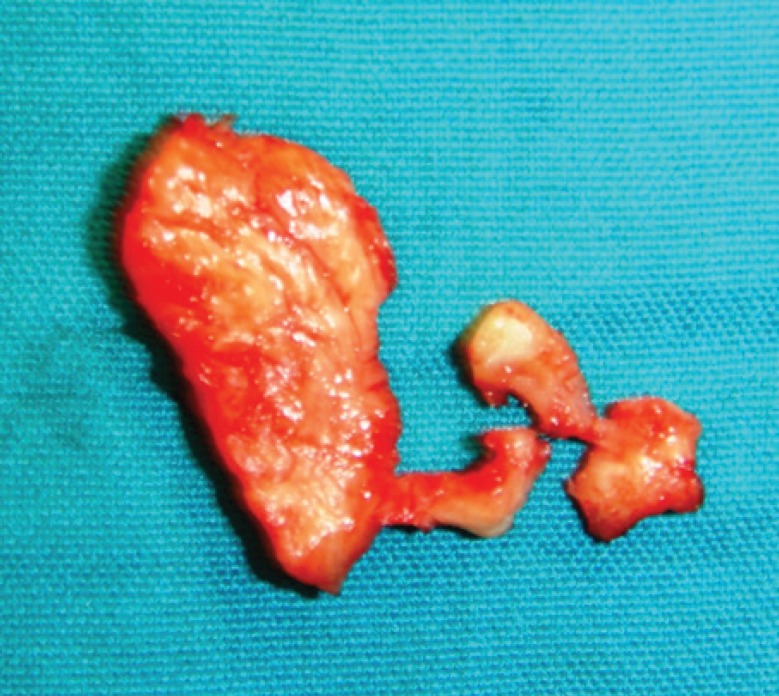
Macroscopic view of the plaque

At a distance of approximately 5 mm from the edge of the plaque and away from the neurovascular bundle, a longitudinal incision was made in tunica albuginea parallel to the lesion. First the plaque was separated from the tissue of the corpus cavernosum through this incision. Then, it was pressed with a finger from the inside to separate it from the tunica albuginea and then cut off with a knife (Figure 3-5). When the plaque was completely removed the edges of the tunica were closed with a continuous suture using 3-0 nylon. 

**Fig. 6:**
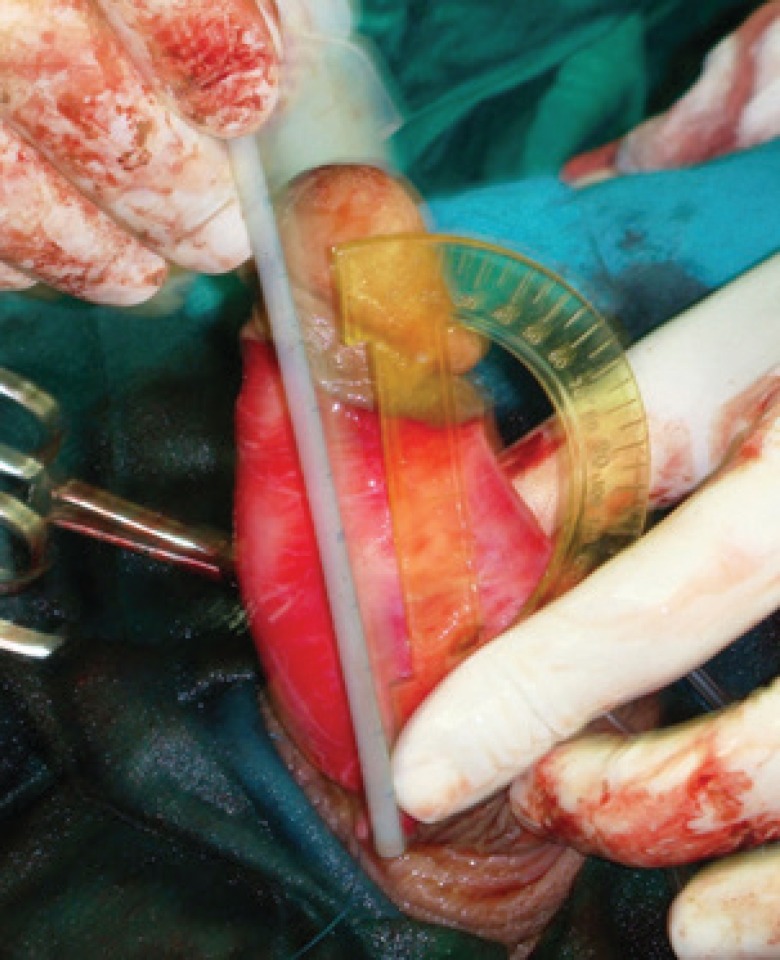
Complete correction of the curvature is documented with an artificial erection after the surgery

An artificial erection was performed after the correction to estimate residual penile curvature and final penile length (Figure 6). A 14 Fr Foley catheter was placed at the end of surgery and was removed after 24 hours. A broad spectrum antibiotic was administered for 5 days. Sexual intercourse was forbidden for 6 weeks. All patients underwent the following investigations periodically within 12 months after the treatment: measurements of penile length and curvature angle in the rigidity phase, and sexual satisfaction. The overall sexual satisfaction was measured by visual scale ranging from 0-10; “very dissatisfied” was considered 0 and “very satisfied” was numbered 10. Data analysis was done by Repeated Measurement test with the help of SPSS software. A *p *value less than 0.05 was considered as significant. 

## RESULTS

The mean (range) age of our patients was 51.4±5.34 (42-59) years. The mean (range) angle of penile curvature was 35±7 (25-45) degrees. The mean (range) operation time was 53.5 (45-60) minutes. No intraoperative complication was observed and all patients were discharged after 1 day. Thirty (86%) patients did not have any penile curvature or they had a curvature up to 10 degrees after this procedure and all of them were able to have normal sexual intercourse. 

In 5 cases (14%) intercourse was impaired only by persistent penile curvature of over 20 degrees. All patients obtained restoration of their previous length without disorders of sensation within the glans penis. The overall subjective sexual satisfaction which measured by visual scale, significantly improved from 4.2±1.7 to 8±1.6. The follow-up of 6 and 12 months after the operation lead to the same results (Table 1).

**Table 1:** Clinical results of the surgery

**Parameter**	**Before Surgery (months)** **n=35**	**After Surgery (months)** **n=35**			***p*** ** value**
		**3 **	**6 **	**12 **	
Penile Curvature (Degree)					
Mean±SD Range	35±7 25-45	8±7 0-30	8±7 0-30	8±7 0-30	<0.001
Penile Length (Cm)					
Mean±SD Range	12.7±1.5 10-15	12.7±1.6 10-15	12.7±1.6 10-15	12.7±1.6 10-15	0.334
Sexual Satisfaction					
Mean±SD Range	4.2±1.7 2-7	8±1.6 3-9	8±1.6 3-9	8±1.6 3-9	<0.001

## DISCUSSION

Peyronie’s disease is a therapeutic challenge to the urologist in clinical practice as ever. The etiology, pathophysiology, and management of disease are remained controversial. Initially, it is better that patients are managed medically, particularly in the acute phase. Surgical treatment is advocated for patients with disabling deformities in the chronic phase of the disease. Surgical management of Peyronie’s disease can be divided into 3 types of (i) Procedures that shorten the convex of uninvolved side of the tunica albuginea (shortening procedures), (ii) Procedures that lengthen the concave of diseased side (lengthening procedures), and (iii) Implantation of penile prosthesis.^4^

Although plication of tunica albuginea or Nesbit operation results in excellent straightening of penis but cause penile shortening and major sensory changes of the glans area that precludes its use in patients with severe deformities, large plaques, or small penises.^5^^-^^7^ For these patients, superior results may be obtained using tunical plaque incision or excision and then covering the defect with graft material (lengthening procedures). In surgical methods that used plaque excision and graft replacing accompanied by disorders of sensation within the glans penis, post-operative erectile dysfunction, and sometimes reduction in penile length and development of cicatricial lesions at the operative site resulting in a recurrent of the disease.^2^^,^^4^^,^^8^

Although penile prosthesis implantation is very successful but in older patients with vascular insufficiency, erectile dysfunction and acquired penile deformity, it is complex technique and results in glans paresthesia and urethral damage.^8^^,^^9^ Smith in 1966 reported that both inflammatory process and presence of a plaque in the later stage of Peyronie’s disease were located between the tunica albuginea and corpus cavernosum.^10^ This opinion was confirmed by the studies of Devine *et al.*,^11^ resulted in introduction of a new operation method (intracavernosal plaque excision) in 2003.

The outcomes of this new operation method have published only on 16 patients.^4^ It seems that Peyronie’s plaques involve the inner circular layer of tunica albuginea and can be removed, while leaving the outer layer intact.^1^ Our surgeries and their results support this hypothesis. The operation is based on a single incision in the tunica albuginea in order to avoid any damage to the neurovascular bundle and then remove the plaque through the same incision without excising the tunica. 

Contrary to other techniques that are used for plaque excision, this method not only eliminated the need to mobilize the neurovascular bundle that which frequently resulted in sense disturbances of glans penis,^4^^,^^10^ but also the use of any graft which in turn effectively prevents penile shortening, delayed healing and the resultant erectile dysfunction. Operation time is much shorter than the other techniques such as incision or excision and patching operations and does not cause reduction in penile length.

The results of surgical treatment of 35 patients showed a near complete straightening of the penis in 30 cases (86%) with restoration of sexual life. In the available literature, we would find only one work for comparison. Darewics et al. in 16 cases showed 12.5% of cases of impaired intercourse but penile shortening, erectile dysfunction and disorders of glans sensation were absent.^4^ We demonstrated that intracavernosal plaque excision is a simple and easy method to perform and as it does not result in penile shortening, loss of sensation or erectile dysfunction, this method can be recommended for appropriate selected patients of Peyronie’s disease.

## CONFLICT OF INTEREST

The authors declare no conflict of interest.
